# Blood Cell Palmitoleate-Palmitate Ratio Is an Independent Prognostic Factor for Amyotrophic Lateral Sclerosis

**DOI:** 10.1371/journal.pone.0131512

**Published:** 2015-07-06

**Authors:** Alexandre Henriques, Hélène Blasco, Marie-Céline Fleury, Philippe Corcia, Andoni Echaniz-Laguna, Laura Robelin, Gabrielle Rudolf, Thiebault Lequeu, Martine Bergaentzle, Christian Gachet, Pierre-François Pradat, Eric Marchioni, Christian R. Andres, Christine Tranchant, Jose-Luis Gonzalez De Aguilar, Jean-Philippe Loeffler

**Affiliations:** 1 INSERM, U1118, Mécanismes Centraux et Périphériques de la Neurodégénerescence, Strasbourg, France; 2 Université de Strasbourg, UMR_S1118, Fédération de Médecine Translationnelle, Strasbourg, France; 3 INSERM, Université François-Rabelais, U930, Neurogénétique et Neurométabolomique, Tours, France; 4 CHRU de Tours, Laboratoire de Biochimie et de Biologie Moléculaire, Tours, France; 5 Hôpitaux Universitaires de Strasbourg, Service de Neurologie, Fédération de Médecine Transrationnelle, Strasbourg, France; 6 CHRU de Tours, Centre SLA, Tours, France; 7 Université de Strasbourg, IPHC, Equipe de Chimie Analytique des Molécules BioActives, Illkirch-Graffenstaden, France; 8 CNRS, UMR 7178, Strasbourg, France; 9 INSERM, Université de Strasbourg, UMR_S949, Etablissement Français du Sang - Alsace, Strasbourg, France; 10 Assistance Publique - Hôpitaux de Paris, Hôpital de la Pitié-Salpêtrière, Fédération des Maladies du Système Nerveux, Centre Référent Maladie Rare SLA, Paris, France; Inserm, FRANCE

## Abstract

Growing evidence supports a link between fatty acid metabolism and amyotrophic lateral sclerosis (ALS). Here we determined the fatty acid composition of blood lipids to identify markers of disease progression and survival. We enrolled 117 patients from two clinical centers and 48 of these were age and gender matched with healthy volunteers. We extracted total lipids from serum and blood cells, and separated fatty acid methyl esters by gas chromatography. We measured circulating biochemical parameters indicative of the metabolic status. Association between fatty acid composition and clinical readouts was studied, including ALS functional rating scale-revised (ALSFRS-R), survival, disease duration, site of onset and body mass index. Palmitoleate (16:1) and oleate (18:1) levels, and stearoyl-CoA desaturase indices (16:1/16:0 and 18:1/18:0) significantly increased in blood cells from ALS patients compared to healthy controls. Palmitoleate levels and 16:1/16:0 ratio in blood cells, but not body mass index or leptin concentrations, negatively correlated with ALSFRS-R decline over a six-month period (*p*<0.05). Multivariate Cox analysis, with age, body mass index, site of onset and ALSFRS-R as covariables, showed that blood cell 16:1/16:0 ratio was an independent prognostic factor for survival (hazard ratio=0.1 per unit of ratio, 95% confidence interval=0.01-0.57, *p*=0.009). In patients with high 16:1/16:0 ratio, survival at blood collection was extended by 10 months, as compared to patients with low ratio. The 16:1/16:0 index is an easy-to-handle parameter that predicts survival of ALS patients independently of body mass index. It therefore deserves further validation in larger cohorts for being used to assess disease outcome and effects of disease-modifying drugs.

## Introduction

Amyotrophic lateral sclerosis (ALS) is a degenerative condition characterized by the loss of upper and lower motor neurons. Most patients initially present with muscular weakness and fasciculations in the limbs while others show dysphagia or dysarthria, indicating an onset in the bulbar area. Disease progresses to generalized paralysis and eventually death, usually occurring from respiratory failure within 1–5 years of diagnosis. Notably, 20% of patients live longer than 5 years, and 10% survive for more than 10 years. Symptoms develop typically between 40–70 years of age. Prevalence is slightly higher in men than in women but becomes comparable with increasing age. Patients with bulbar onset have more rapid progression and shorter life expectancy than patients with limb onset. Younger patients have longer disease duration than older ones [1–3].

According to the revised El Escorial and the Awaji criteria [4, 5], a progressive spread of symptoms involving upper and lower motor neurons is required for a definitive diagnosis of ALS. At onset, however, symptoms manifest partially, and the verification of such criteria often takes more than one year. Disease progression is mostly assessed by consensual clinical scores, the most used of which is the ALS functional rating scale-revised (ALSFRS-R) [5]. This scale is employed to evaluate the pace of disease progression, predict survival and assess the effects of disease-modifying drugs in therapeutic trials. However, it depends on the interpretation of questionnaires on the patients’ quality of life and hence suffers from unsufficient sensitivity. There is therefore an undeniable need for robust biological markers that help to diagnose ALS shortly, and prognosticate disease outcome accurately.

ALS is characterized by important alterations in energy homeostasis [6, 7]. Hypermetabolism, abnormal glucose tolerance and dyslipidemia have a high incidence in ALS patients and persist over time [8–13]. Since these modifications can be easily monitored with minimally invasive impact, new biomarkers were proposed based on metabolic readouts. The results were however conflicting. Using univariate analysis, high LDL/HDL cholesterol ratio or elevated content of total cholesterol and triglycerides, was associated with better prognosis in French, German, Dutch and North American cohorts [12–15] or with slower disease progression in an Italian cohort [16]. Based on multivariate analysis, BMI at diagnosis remains the only metabolic independent prognostic factor for survival in ALS [13, 14].

Overall energetic metabolism influences the degree of unsaturation and length of fatty acids present in lipids. The endogenous conversion of palmitate into palmitoleate and of stearate into oleate is catalyzed by Δ9 stearoyl-CoA desaturase (SCD), a lipogenic enzyme highly sensitive to global energetic homeostasis. Palmitoleate to palmitate (16:1/16:0) and stearate to oleate (18:1/18:0) ratios are used as proxies of nutritional status in conditions such as obesity and cancer [17, 18]. Here we hypothesized that the changes in the proportions of fatty acids from blood lipids are a source of novel, reliable markers to help monitoring disease severity in ALS patients. We analyzed chromatographic profiles of fatty acids derived from total lipids in serum and clotted blood cells. We compared fatty acid composition between patients and healthy controls. We also correlated changes in patients’ fatty acid composition with clinical and biochemical parameters. We used multivariate Cox proportional hazards model analysis to assess whether the detected changes predict patients' life expectancy.

## Material and Methods

### Standard protocol approvals, registrations, and patient consents

Blood samples were collected during routine clinical evaluation for ALS patients and during blood donation for healthy controls. Patients have received appropriate information about the use of their blood samples in medical research and gave oral informed consent to clinicians (Tours) or gave written informed consent (Strasbourg). Signed or documented consent was not requested since study includes only blood collected during routine procedure. The ethical committees of the hospitals in Tours (Comité de protection des personnes "Ouest I") and Strasbourg (Comité de protection des personnes "Est-IV") approved the consent procedures and protocol (CPPRB 09/40–n°AC-2008-438/n°DC-2009-1002).

### Patients and control subjects

This is a case-control study performed with 117 ALS patients and 48 healthy controls enrolled between April 2011 and September 2014. Patients were recruited from two referral centers for ALS located in Strasbourg (Eastern France) and Tours (Western-Central France). Patients diagnosed as having probable or definite ALS, according to the revised El Escorial criteria, were included [5]. According to typical epidemiology, patients with a familial history of ALS represented about 10% [19]. Healthy volunteers were recruited from the Etablissement Français du Sang (Strasbourg, France), and were matched for gender and age. Blood samples were collected under non-fasting conditions, since the metabolic status of ALS patients in response to fasting may differ from that of controls [8]. Individuals taking medications for treatment of diabetes or related metabolic conditions were excluded [14]. Age, gender and body mass index (BMI, calculated as weight in kg divided by height in m^2^) were recorded at blood collection. The site of onset was defined as bulbar or spinal (*i*.*e*., limb). Disease onset was designated as the time of initial motor deficit. Survival was the interval between the point of blood collection and death from confirmed ALS related complications. Disease duration was the interval between onset of symptoms and death. ALSFRS-R scores were recorded at blood collection. To determine the rate of ALSFRS-R decline, scores were also registered six months later, except for a minority of cases where scores were obtained six months before the collection time. The decline was calculated as the loss of points of ALSFRS-R over six months.

### Blood sample preparation and gas chromatography

Blood samples were collected from venous punction in red top tubes and allowed to clot for 30 minutes at room temperature. After centrifugation at 4000g for 10 minutes at 4°C, serum and clot were separately snap frozen on dry ice and kept at -80°C until processing. In addition to serum, we also analyzed clotted blood cells, since lipid turnover in this membrane-enriched fraction ranges from five to fifteen days, and hence fatty acid composition is hardly affected by food intake prior to collection [20–22]. Total lipids were extracted following a modified version of the Bligh and Dyer method, as previously described [23]. After separation from the parent lipid molecules, the obtained fatty acids were converted into fatty acid methyl ester derivatives and stored at -20°C until analysis. Gas chromatography was performed using a Varian 3400CX chromatograph fitted with a WCOT fused silica capillary column of 100 m x 0.25 mm x 0.20 μm, coated with polar highly substituted cyanopropyl CP-SIL 88 phase, as described in detail [24]. Results were expressed as relative percentages of the total fatty acids detected.

### Quantification of biochemical parameters

Circulating levels of triglycerides, total cholesterol and free fatty acids were determined with TR210, CH200 and FA115 Randox kits, respectively, following standard protocols according to manufacturer’s recommendations (Laboratoires Randox, Roissy, France). Leptin levels were determined using a commercial OKAA00022 ELISA kit, according to manufacturer’s instructions (Aviva Systems Biology, San Diego, CA).

### Statistical analysis

Prism version 6.0 (GraphPad, San Diego, CA) and JMP v11.0.0 (SAS, Grégy-sur-Yerres, France) were used for statistical analysis. Unless otherwise indicated, data are expressed as the mean±SEM. Normality was assessed by d’Agostino-Pearson omnibus test. Comparisons between fatty acid profiles were assessed by multiple unpaired two tailed *t*-test, with false discovery rate set to 10%. Differences in fatty acid ratios between groups were analyzed by one-way ANOVA followed by Fisher’s least significant difference test, or by Kruskal-Wallis test followed by Dunn’s multiple comparisons test. Receiver-operator characteristic (ROC) curves were used to study sensitivity and specificity of SCD indices when discriminating between ALS patients and controls. Non-parametric Mann-Withney test was used to assess differences in circulating biochemical parameters. Significant correlation between two variables was determined by non-parametric Spearman test. Multivariate regression analysis was performed using Cox proportional hazard models. The results are reported as hazard ratios with 95% confidence intervals. Hazard ratios are per change in regressor over entire range for continuous variable. Kaplan-Meier curves were used to visualize survival rates after univariate analysis by Gehan-Breslow-Wilcoxon test. Significance level was set at *p*<0.05. Experimenters were blinded until study endpoint.

## Results

### Palmitoleate and oleate levels and stearoyl-CoA desaturase indices are increased in ALS patients

In a first step to identify biomarkers that could reflect metabolic changes in ALS and help predict its outcome, we performed a case-control study on the fatty acid composition of blood lipids extracted from 48 ALS patients and age- and gender-matched healthy controls (Table 1).

**Table 1:** Clinical and biochemical characteristics of ALS patients and control subjects. ALSFRS-R, ALS functional rating scale-revised; BMI, body mass index.

ITEMS	Control	ALS	
Cohort size	48	48	
Gender ratio (F/M)	18/30	18/30	
Age at collection (years)	54.1 [31.7–66.6]	54.9 [34.9–68.7]	
Duration (days)	N/A	809 ± 75.3	
ALSFRS-R (score)	N/A	29.8 ± 1.3	
Site at onset (bulbar/spinal)	N/A	14/35	
BMI (kg/m²)	N/A	23.6 ± 0.6	
Triglycerides (g/L)	1.24 ± 0.1	1.44 ±0.1	
Total cholesterol (g/L)	2.60 ± 0.2	2.54 ± 0.2	
Free fatty acid (mM)	0.12 ± 0.0	0.27 ± 0.1	*
Leptin (ng/mL)	9.66 ± 1.5	6.11 ± 0.9	*

*p<0.05 (Mann-Withney test).

Chromatographic profiles revealed up to 18 different fatty acids that were classified into saturated, monounsaturated and polyunsaturated fatty acids, according to the number of carbon-carbon double bonds in the aliphatic chain. In general, proportions of several members from each class showed significant differences between ALS patients and healthy subjects, which were more marked in blood cell pellets than in serum (Table 2). Changes in polyunsaturated fatty acids may reflect altered susceptibility to lipid peroxidation and/or the presence of an inflammatory process. Lipid peroxidation susceptibility is commonly assessed by the peroxidability index (PI), which is based on the number of unsaturations present in the fatty acid chain [25]. Inflammatory processes can be estimated by the arachidonate (20:4n-6) to eicosapentaenoate (20:5n-3) ratio (ARA/EPA index) [26, 27]. In our study, PI was 16% lower in the blood cell fraction of ALS patients as compared to controls (S1 Fig), whereas ARA/EPA index was not significantly modified (S1 Fig).

**Table 2:** Fatty acid composition of total lipids in serum and blood cells from ALS patients and control subjects. FA, fatty acid; SFA, saturated fatty acids; MUFA, monounsaturated fatty acids; PUFA, polyunsaturated fatty acids.

	Serum			Blood pellet		
% of FA	Control (n = 48)	ALS (n = 48)		Control (n = 48)	ALS (n = 48)	
**14:0**	1.27 ± 0.1	1.22 ± 0.1		0.58 ± 0.0	0.80 ± 0.1	**
**16:0**	27.33 ± 0.3	26.27 ± 0.3	*	23.54 ± 0.2	24.60 ± 0.5	*
**18:0**	9.78 ± 0.2	9.46 ± 0.2		15.28 ± 0.2	13.77 ± 0.3	***
**20:0**	0.014 ± 0.007	0.013 ± 0.003		0.052 ± 0.006	0.061 ± 0.007	
**22:0**	0.18 ± 0.014	0.13 ± 0.010	***	0.05 ± 0.008	0.06 ± 0.008	
**16:1n-7**	1.82 ± 0.12	1.73 ± 0.11		0.93 ± 0.07	1.18 ± 0.08	*
**18:1n-9**	23.97 ± 0.4	26.79 ± 0.6	***	19.10 ± 0.3	22.59 ± 0.5	***
**20:1n-9**	0.15 ± 0.013	0.20 ± 0.033		0.24 ± 0.006	0.27 ± 0.010	**
**22:1n-9**	0.001 ± 0.001	0.005 ± 0.001		0.011 ± 0.002	0.016 ± 0.003	
**18:2n-6**	22.47 ± 0.5	21.21 ± 0.5		16.97 ± 0.3	17.64 ± 0.4	
**18:3n-6**	0.26 ± 0.014	0.29 ± 0.021		0.18 ± 0.014	0.24 ± 0.021	*
**20:2n-6**	0.24 ± 0.007	0.25 ± 0.008		0.24 ± 0.004	0.26 ± 0.008	*
**20:3n-6**	1.93 ± 0.1	1.78 ± 0.1		1.87 ± 0.1	1.71 ± 0.1	
**20:4n-6**	7.29 ± 0.2	6.96 ± 0.3		14.21 ± 0.2	11.16 ± 0.4	**
**18:3n-3**	0.44 ± 0.028	0.67 ± 0.120		0.24 ± 0.015	0.42 ± 0.061	**
**20:3n-3**	0.007 ± 0.002	0.016 ± 0.003	*	0.011 ± 0.002	0.015 ± 0.003	
**20:5n-3**	0.92 ± 0.07	0.80 ± 0.07		0.98 ± 0.05	0.84 ± 0.07	
**22:5n-3**	0.21 ± 0.01	0.23 ± 0.02		2.14 ± 0.07	1.31 ± 0.08	***
**22:6n-3**	1.71 ± 0.09	1.99 ± 0.09	*	3.37 ± 0.13	3.05 ± 0.15	
**Σ SFA**	38.57 ± 0.4	37.08 ± 0.4	**	39.51 ± 0.2	39.24 ± 0.6	
**Σ MUFA**	25.95 ± 0.5	28.72 ± 0.6	**	20.28 ± 0.3	24.10 ± 0.5	***
**Σ PUFA**	35.48 ± 0.6	34.20 ± 0.6		40.22 ± 0.3	36.66 ± 0.8	***

*p<0.05,

**p<0.01,

***p<0.001 (multiple unpaired two tailed *t*-test).

**Fig 1:**
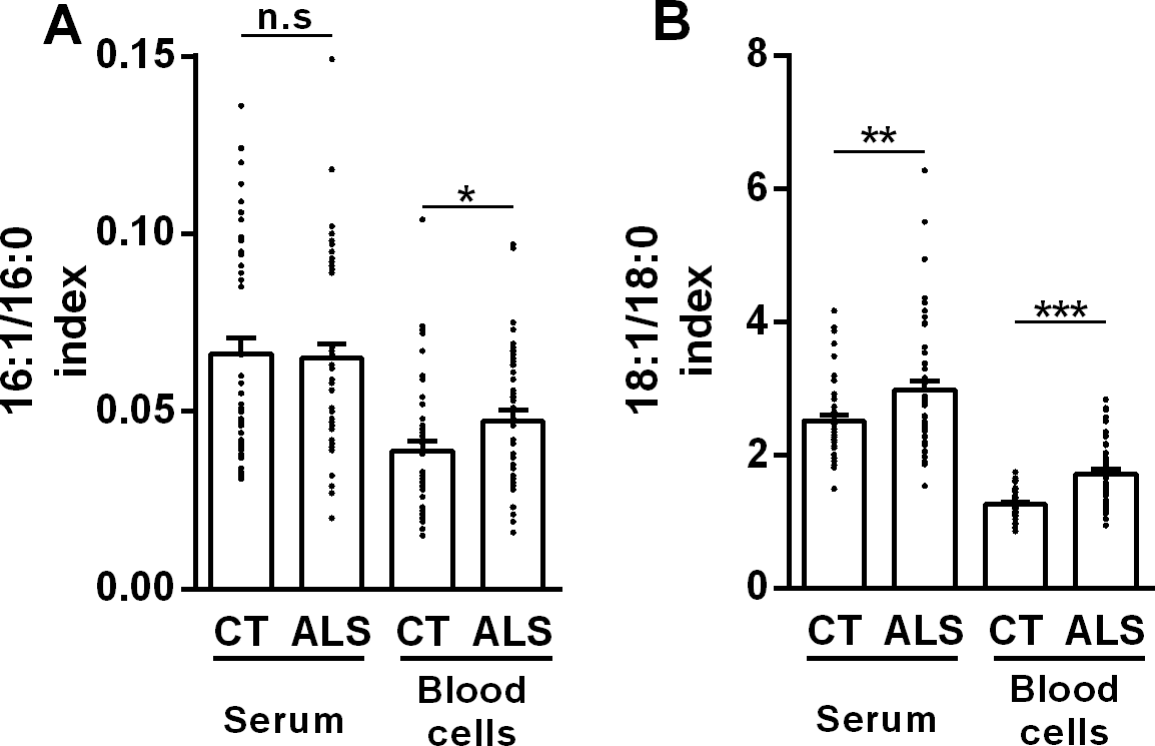
SCD indices are higher in ALS patients than in control subjects. (A) Palmitoleate to palmitate ratio (16:1/16:0) in serum and blood cells from ALS patients (ALS) and control subjects (CT). (B) Oleate to stearate ratio (18:1/18:0) in serum and blood cells from ALS patients (ALS) and control subjects (CT). *p<0.05, **p<0.01, ***p<0.001 (Mann-Withney test, n = 48).

Notably, the proportion of monounsaturated fatty acids was strongly increased in ALS patients. In particular, palmitoleate and oleate levels increased significantly in blood cells, and higher oleate levels were also observed in serum (Table 2). Palmitoleate and oleate are synthetized by SCD from palmitate and stearate, so that the respective product to substrate ratios are considered as an index of SCD activity [17, 18]. SCD indices 18:1/18:0 and 16:1/16:0 appeared significantly higher in ALS blood cell pellets than in controls. Oleate to stearate ratio was also increased in patients' sera (Fig 1A and 1B).

To assess whether these changes had diagnostic value, we performed ROC analysis. Serum and blood cell pellet 18:1/18:0 ratio, as well as blood cell pellet 16:1/16:0 ratio, showed areas under the curves that were significant between ALS and control groups. Nevertheless, sensitivity and specificity scores based on these ratios were insufficient to distinguish between those individuals with the disease and those without the disease (S2 Fig).

High blood SCD index has been typically associated with conditions characterized by enhanced lipogenesis, such as obesity and insulin resistance [28]. Thus, we determined whether our ALS patients differed from healthy controls by a higher degree of dyslipidemia, as previously reported in other studies [12, 13]. Contents of triglycerides and total cholesterol in patients were comparable to those found in control subjects. Leptin levels, however, appeared significantly decreased, suggesting lower fat reserves [29]. Finally, circulating free fatty acids were more abundant in ALS patients, suggesting increased lipid breakdown by adipocytes [30] (Table 1). In all, this metabolic trait, indicative of lower adipose tissue stores, paradoxically coexisted with high SCD indices, which are rather characteristic of enhanced lipogenesis.

### Blood cell 16:1/16:0 ratio and palmitoleate levels correlate with disease progression

**Fig 2:**
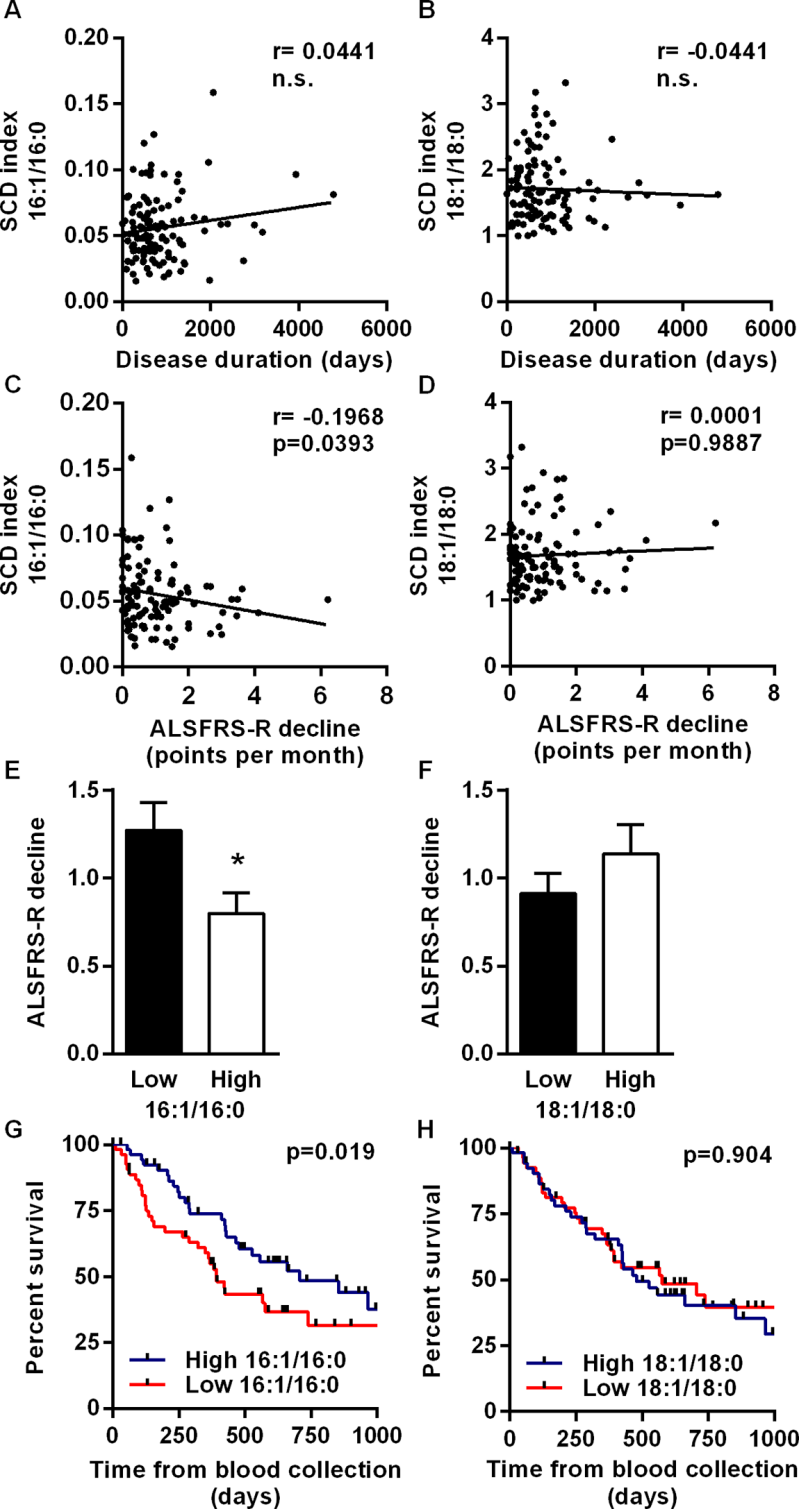
Blood cell 16:1/16:0 ratio correlates with disease progression and affects survival of ALS patients. Correlation between 16:1/16:0 and 18:0/18:1 ratios and disease duration (A, E) at blood collection or ALSFRS-R slope (B, F), determined as the decline of the score over a period of six months starting at the point of blood collection. Correlation coefficients (r) and *p*-values are indicated. n.s., non-significant *p*-value (Spearman test, n = 111). Based on the median ratio of the population, ALSFRS-R decline is shown in patients with low or high 16:1/16:0 ratio (C), and in patients with low or high 18:1/18:0 ratio (G). *p<0.05 (Mann-Withney test). (D, H) Kaplan-Meier curves of survival in the subgroups of patients as above. Survival was the interval between the point of blood collection and death. *P*-values are indicated (Gehan-Breslow-Wilcoxon test, n = 117).

In a second step to identify biomarkers that could help to prognosticate ALS evolution, we performed a cross-section study with 117 ALS patients, including the previous 48 ones (S1 Table). We focused on SCD indices in blood cell pellets, since differences in this lipid fraction between patients and controls were more robust. None of the SCD indices correlated with the duration of the disease from symptom onset to time of blood collection (Fig 2A and 2B), suggesting the absence of interferences due to the variability in the time of sampling. In contrast, 16:1/16:0 ratio showed a significant negative correlation with disease progression (Fig 2C and 2D), which was defined as the loss of ALSFRS-R score over a period of six months (unit loss/month) [31]. In fact, levels of palmitoleate itself were also significantly associated with the decline of ALSFRS-R (r = -0.1996, p<0.05). The relationship between SCD index and disease progression was also apparent when patients were divided into two subsets of low and high 16:1/16:0 ratio, based on the median of the population. ALSFRS-R declined more slowly in patients with high 16:1/16:0 ratio than in patients with low 16:1/16:0 ratio. Other measured indices, including 18:1/18:0 ratio, PI and ARA/EPA ratio, were not significantly associated with the rate of disease progression (Fig 2E and 2F, data not shown). However, both SCD indices in blood cells correlated negatively with PI, indicating that a higher proportion of monounsaturated fatty acids is associated with a lower susceptibility to lipid peroxidation (S1 Fig).

### Blood cell 16:1/16:0 ratio and palmitoleate levels are independent prognostic factors for survival

The findings mentioned above prompted us to investigate whether blood cell pellet 16:1/16:0 ratio or palmitoleate could predict life expectancy. At the time of study completion (September 30^th^ 2014), 57 out of 117 patients were alive or with unknown status, and were censored. Univariate analysis revealed that age, site of onset, BMI, disease duration and ALSFRS-R were significantly associated with survival, defined as the interval between the point of blood collection and the fatal event. Gender and ALS center did not reach significance (S2 Table). Then we performed multivariate Cox regression analysis adjusted for these covariates, except for gender and ALS center. We found that higher 16:1/16:0 ratios were significantly associated with better survival rates (HR = 0.09, 95%CI = 0.01–0.57, *p* = 0.009) (Table 3). Similar results were obtained when patients were classified as having low or high 16:1/16:0 ratio based on the median of the population (HR = 0.58, 95%CI = 0.34–0.99, *p* = 0.0483).

**Table 3:** Analysis of the survival of ALS patients using multivariate Cox proportional hazards model.

Variable	Items	Risk ratio	Lower 95%	Upper 95%	Prob>Chisq
Site of onset	Spinal	0.442	0.25	0.80	0.0076
	Bulbar	2.264	1.25	4.06	
Age	-	4.63	1.20	19.5	0.0257
Disease duration	-	0.28	0.03	1.93	0.2132
BMI	-	0.15	0.01	1.22	0.077
ALSFRS-R	0–35	1 (reference)	-	-	-
	36–39	0.529	0.26	0.99	0.0478
	40–42	0.240	0.04	0.83	0.0206
	>42	0.247	0.04	0.86	0.0257
SCD 16:1/16:0	-	0.09	0.01	0.57	0.0090

The association of 16:1/16:0 ratio with the survival of ALS patients (n = 117) was analyzed by adjusting for site of onset, age, BMI and ALSFRS-R. Survival was the interval between the point of blood collection and death. ALSFRS-R was categorized into four groups, according to Gordon et al. [32]. BMI, body mass index; ALSFRS-R, ALS functional rating scale-revised; SCD, Δ9 stearoyl-CoA desaturase.

Kaplan–Meier curves showed that patients with a high 16:1/16:0 ratio, based on the median of the population, had a prolonged life expectancy of almost 11 months (median survival of 23.6 months, *p* = 0.019), as compared to patients with low 16:1/16:0 ratio (median survival of 13.1 months) (Fig 2G). Higher levels of palmitoleate itself were also significantly associated with extended life span (HR = 0.11, 95%CI = 0.015–0.64, *p* = 0.0141). The 18:1/18:0 ratio was not associated with survival (Fig 2H, and data not shown). In all, our results suggest that 16:1/16:0 ratio is a new and robust prognostic factor, independent from BMI, for survival of ALS patients.

### BMI and leptin levels are not independent prognostic factors for survival

**Fig 3:**
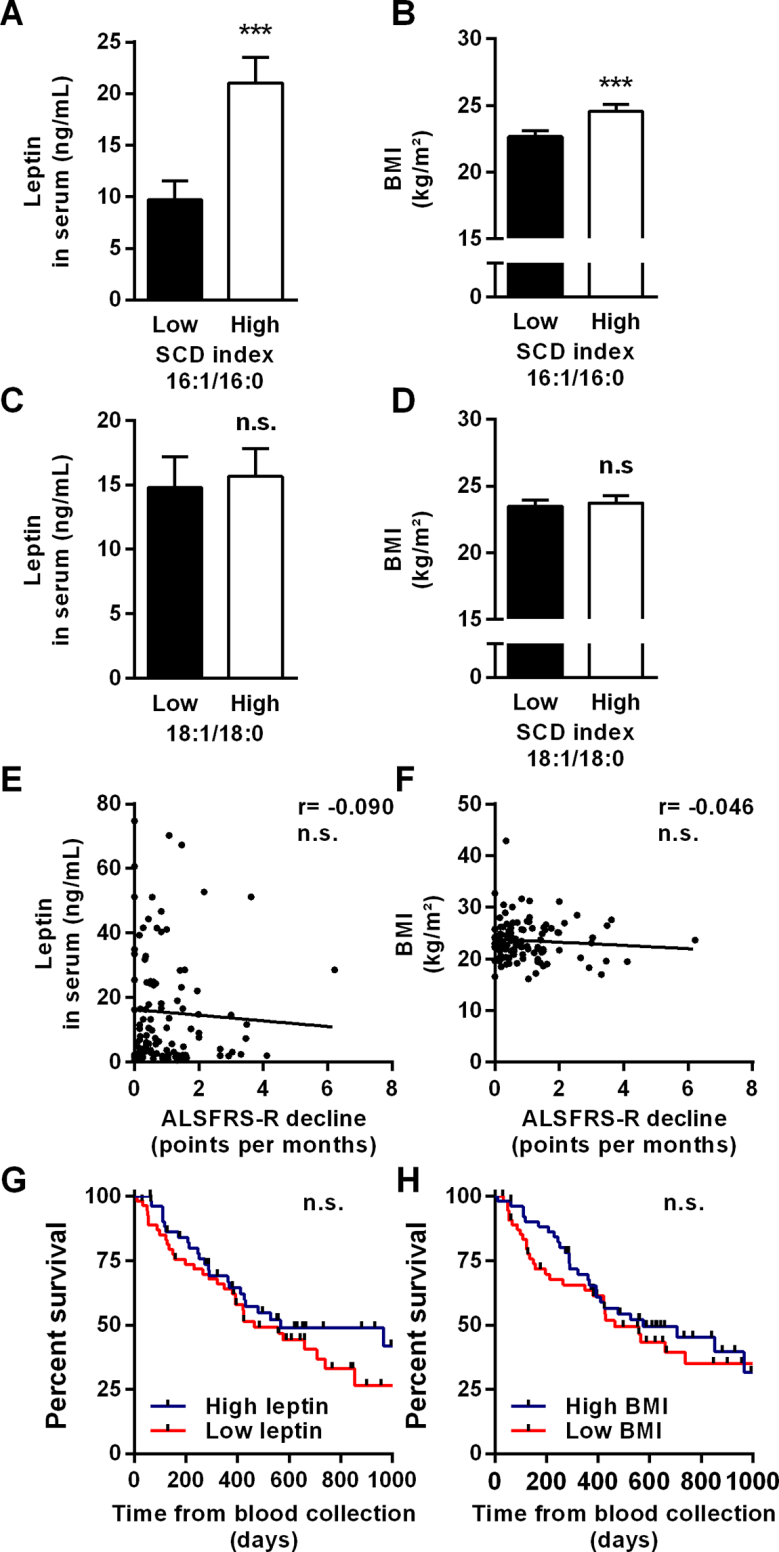
Blood cell 16:1/16:0 ratio is associated with leptin levels and BMI. Circulating amounts of leptin (A, C) and BMI (B, D) in patients with low or high 16:1/16:0 ratio, and in patients with low or high 18:1/18:0 ratio (Mann-Withney test, n = 117). (E, F) Correlation between leptin levels (A) or BMI (C) and ALSFRS-R slope, determined as the decline of the score over a period of six months starting at the point of blood collection. Correlation coefficients (r) are indicated. n.s., non-significant *p*-value (Spearman test, n = 111). (G, H) Kaplan-Meier curves of survival in patients with low or high leptin levels, and in patients with low or high BMI, according to the median values of the population. Survival was the interval between the point of blood collection and death. n.s., non-significant *p*-value (Gehan-Breslow-Wilcoxon test, n = 117).

High SCD index is often associated with metabolic conditions characterized by high BMI. Therefore, we asked whether other markers of global adiposity, such as BMI and leptin concentration [29], measured at the same time as the 16:1/16:0 ratio, could predict survival. Patients with a 16:1/16:0 ratio above the median of the ALS population had significantly higher leptin levels and BMI than patients with lower 16:1/16:0 ratios (Fig 3A and 3B). No differences in BMI or leptin concentrations were observed between patients when considering the 18:1/18:0 ratio (Fig 3C and 3D). Despite the relationship between 16:1/16:0 ratio and such fat tissue proxies, BMI or circulating leptin content did not correlate with ALSFRS-R decline, nor prognosticated survival based on multivariate analysis (Fig 3E–3H). Only when using univariate analysis, BMI was able to predict survival (HR = 0.12, 95%CI = 0.02–0.78, *p* = 0.025).

## Discussion

During the last decade, many efforts have been dedicated to the understanding of the remarkable alterations affecting lipid metabolism in ALS [6, 12]. Several systemic modifications, otherwise considered as important cardiovascular risk factors, such as high LDL/HDL cholesterol ratio [12, 15], or high levels of triglycerides and total cholesterol [13, 14], were shown to be associated with longer life span. Conversely, low LDL/HDL cholesterol ratio and malnutrition correlated with worsening of the respiratory function and shorter survival [16, 33, 34]. None of these metabolic readouts, however, reached statistical significance in multivariate analysis when BMI was included as a confounder variable. High BMI was indeed the only metabolic proxy that was associated with slower disease progression and longer life expectancy in multivariate models [13, 14, 33–35]. In this study, we report that blood lipids from ALS patients have a higher proportion of monounsaturated fatty acids and a lower proportion of polyunsaturated fatty acids. Most importantly, we show that the ratio of palmitoleate to palmitate (16:1/16:0) in the composition of lipids extracted from blood cells can predict life expectancy of ALS patients at the time of blood collection better than other previously proposed metabolic biomarkers, independently of age, site of onset, disease status and, most importantly, BMI. Patients with higher 16:1/16:0 ratio had lower lipid peroxidability and better survival rates, and their disease progressed slower, at least for a period of six months.

Several of the significant results we obtained with the 16:1/16:0 ratio were paralleled by palmitoleate in its own. However, neither 18:1/18:0 ratio nor oleate were able to prognosticate disease outcome. The abundance of oleate in modern diets [36] could interfere with the estimation of its endogenous synthesis and therefore lower the value of this fatty acid as a reliable predictor. In contrast, palmitoleate is a minor fatty acid present in the diet, and hence the 16:1/16:0 ratio is rather the reflect of its endogenous synthesis, which likely allowed a more accurate, independent follow-up of 16:1/16:0 ratio alterations in relation to the course of the disease.

In our multivariate model, the 16:1/16:0 ratio prognosticated life expectancy better than BMI. Notably, BMI failed to predict life span in this model. In line with these findings, the circulating levels of leptin, which represents a more direct measure of body adiposity [29], did not show any association with disease progression or survival. It is tempting to speculate that several uncontrolled parameters, such as inherited susceptibility, lifestyle and nutritional habits, would have shaped BMI, at least in our cohort of patients, long before disease in a way that biased its capacity to predict survival. In contrast to BMI, the 16:1/16:0 ratio reflects the relatively recent incorporation of these fatty acids into lipid species and therefore it may represent better the ongoing metabolism at the time of blood collection. In addition, the 16:1/16:0 ratio measured in blood cell lipids mainly reflects the fatty acid composition of structural membrane phospholipids, which is different from that of energetic triglycerides accumulated in fat pads and largely contributing to establish BMI. These specificities may have contributed to the robustness of the 16:1/16:0 ratio in predicting life expectancy.

High SCD indices have been usually associated with obesity and related metabolic disorders including diabetes [17]. However, our ALS patients were not obese, as deduced from their BMI scores and circulating leptin concentrations, according to commonly accepted criteria [14]. Aside from obesity, SCD activity has also been shown to be stimulated in conditions with atypical energetic requirements, such as anorexia and intense physical training [37–41]. These conditions are often accompanied by recurrent episodes of energy shortage. Such a situation can be experimentally reproduced in rats submitted to relatively long periods of food restriction and subsequent refeeding. SCD expression after refeeding was shown to be increased in the liver of these animals as a means to rapidly store highly energetic lipids for being used later [42]. ALS patients frequently present with hypermetabolism of unknown origin [8, 10]. Therefore, high SCD activity in these patients could be interpreted as a physiological response to the energetic demand imposed by the hypermetabolic trait [8–10]. In this scenario, high lipogenic SCD indices, as those observed in our cohort of patients, could coexist with elevated concentrations of circulating free fatty acids deployed to cope with the energetic requirements. In this respect, our previous studies demonstrated that feeding hypermetabolic mutant SOD1 mice, a well-known animal model for ALS, with a highly caloric fat-enriched diet not only restored lost fat pads but also preserved motor function, protected motor neurons and extended life expectancy [6, 43]. More recent studies performed on another related ALS transgenic mouse line also showed the neuroprotective benefit of a diet enriched in monounsaturated fatty acids, including palmitoleate and oleate [44]. The effects of diets with an enriched content in lipids or carbohydrates have also been evaluated in humans. These yet pilot studies reported benefit in terms of body mass stabilization and lower incidence of adverse events. Due to small cohorts of patients, no conclusions could be formally drawn about the potential survival effects of such highly caloric diets. Nevertheless, they provided proof-of-concept evidence that manipulating nutritional/energetic requirements can be beneficial to ALS patients [45, 46].

There are no well-established biological markers of prognostic value for ALS. Here we built on the *metabolic pathology* of the disease to propose the ratio of palmitoleate to palmitate in the composition of lipids as an independent factor to predict life expenctancy. This parameter could be used in clinical practice and in the design of clinical trials.

## Supporting Information

S1 Fig Peroxidability index and ARA/EPA index. (PPTX)

S2 Fig Receiver-operator characteristic analysis of blood SCD indices. (PPTX)

S1 Table Demographic and clinical characteristics of ALS patients in the prognosis study. (XLSX)

S2 Table Analysis of the survival of ALS patients using univariate Cox proportional hazards model. (XLSX)
