# Supplementary material for: Blood Cell Palmitoleate-Palmitate Ratio Is an Independent Prognostic Factor for Amyotrophic Lateral Sclerosis
Source: PLoS One. 2015 Jul 6;10(7):e0131512. doi: 10.1371/journal.pone.0131512 (PMC4492495; doi:10.1371/journal.pone.0131512)
Supplement: S1 Fig — (PPTX) [file pone.0131512.s001.pptx]

## Slide 1
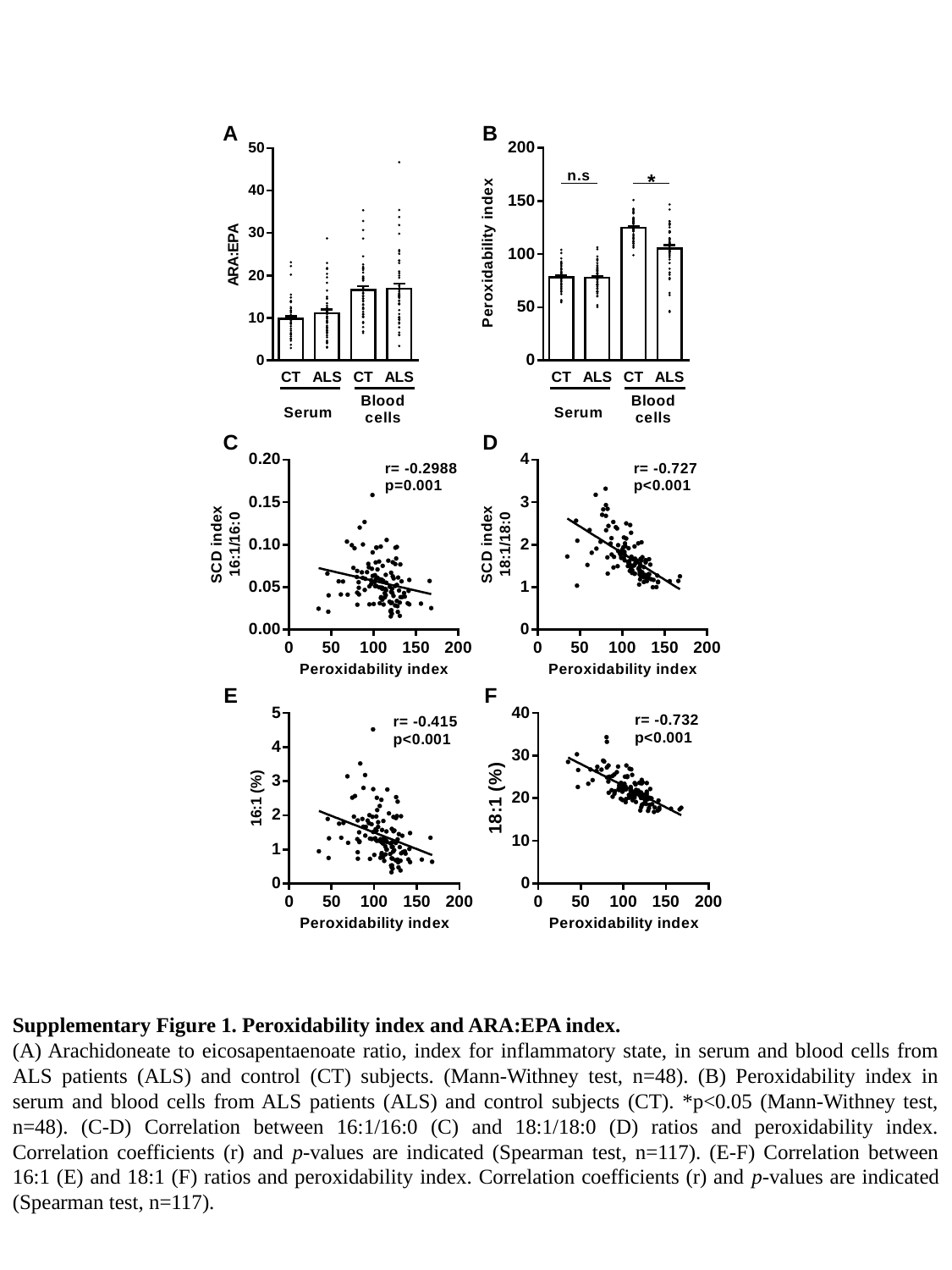

Supplementary Figure 1. Peroxidability index and ARA:EPA index.
(A) Arachidoneate to eicosapentaenoate ratio, index for inflammatory state, in serum and blood cells from ALS patients (ALS) and control (CT) subjects. (Mann-Withney test, n=48). (B) Peroxidability index in serum and blood cells from ALS patients (ALS) and control subjects (CT). *p<0.05 (Mann-Withney test, n=48). (C-D) Correlation between 16:1/16:0 (C) and 18:1/18:0 (D) ratios and peroxidability index. Correlation coefficients (r) and p-values are indicated (Spearman test, n=117). (E-F) Correlation between 16:1 (E) and 18:1 (F) ratios and peroxidability index. Correlation coefficients (r) and p-values are indicated (Spearman test, n=117).
