# Supplementary material for: Blood Cell Palmitoleate-Palmitate Ratio Is an Independent Prognostic Factor for Amyotrophic Lateral Sclerosis
Source: PLoS One. 2015 Jul 6;10(7):e0131512. doi: 10.1371/journal.pone.0131512 (PMC4492495; doi:10.1371/journal.pone.0131512)
Supplement: S2 Fig — (PPTX) [file pone.0131512.s002.pptx]

## Slide 1
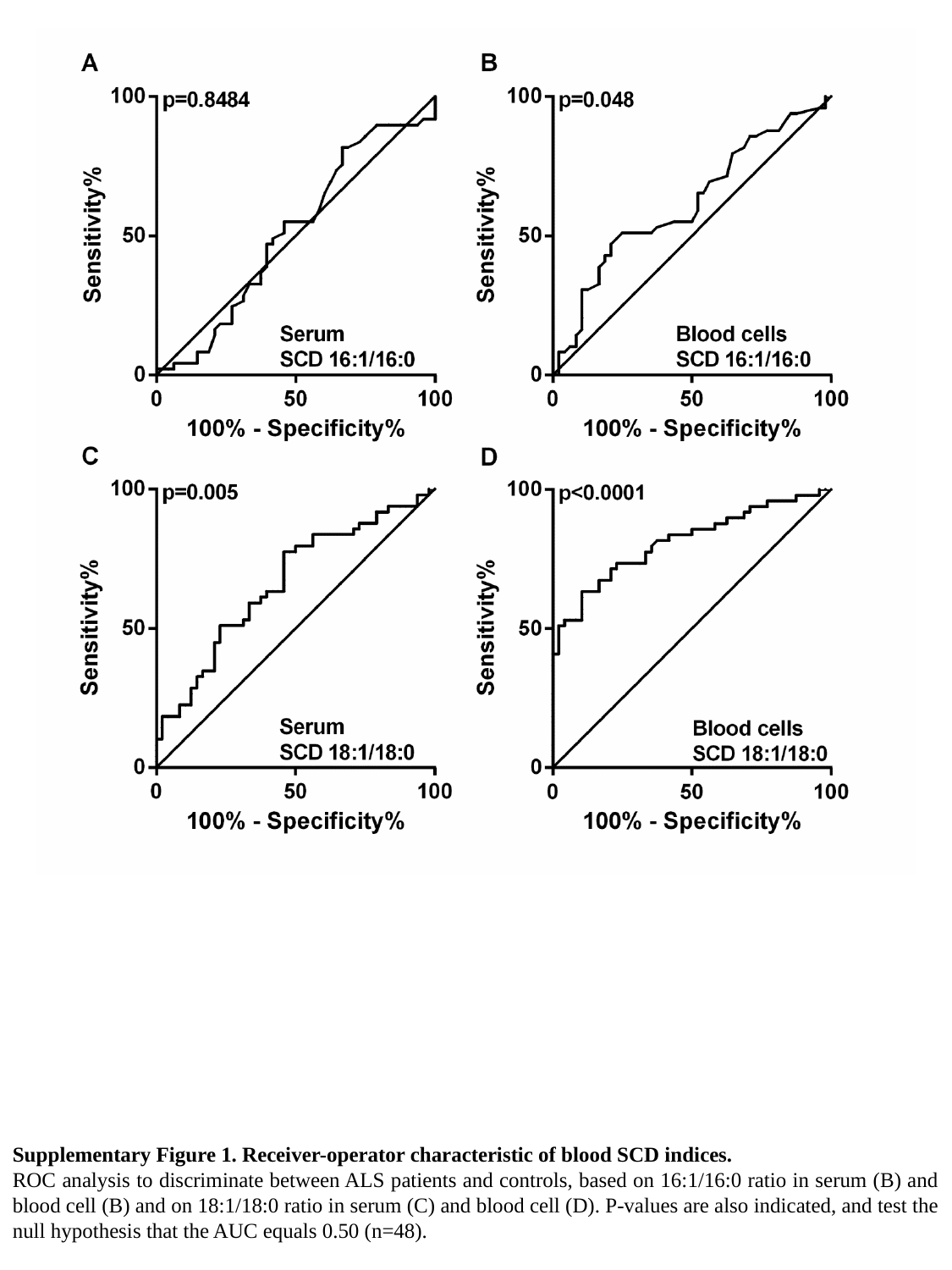

Supplementary Figure 1. Receiver-operator characteristic of blood SCD indices.
ROC analysis to discriminate between ALS patients and controls, based on 16:1/16:0 ratio in serum (B) and blood cell (B) and on 18:1/18:0 ratio in serum (C) and blood cell (D). P-values are also indicated, and test the null hypothesis that the AUC equals 0.50 (n=48).
